# Editorial: Uncovering the Function of the Mitochondrial Protein VDAC in Health and Disease: From Structure-Function to Novel Therapeutic Strategies

**DOI:** 10.3389/fonc.2017.00320

**Published:** 2017-12-22

**Authors:** Varda Shoshan-Barmatz, Hanna Kmita, John J. Lemasters

**Affiliations:** ^1^Department of Life Sciences, The National Institute for Biotechnology in the Negev, Ben-Gurion University of the Negev, Beersheba, Israel; ^2^Institute of Molecular Biology and Biotechnology, Faculty of Biology, Adam Mickiewicz University, Poznań, Poland; ^3^Center for Cell Death, Injury and Regeneration, Department of Drug Discovery and Biomedical Sciences, Medical University of South Carolina, Charleston, SC, United States; ^4^Center for Cell Death, Injury and Regeneration, Department of Biochemistry and Molecular Biology, Medical University of South Carolina, Charleston, SC, United States

**Keywords:** voltage-dependent anion channel protein, mitochondria, metabolism, apoptosis, cancer, neurodegeneration

**Editorial on the Research Topic**

**Uncovering the Function of the Mitochondrial Protein VDAC in Health and Disease: From Structure-Function to Novel Therapeutic Strategies**

This Research Topic on “Uncovering the Function of the Mitochondrial Protein VDAC in Health and Disease: From Structure-Function to Novel Therapeutic Strategies” involves five papers and focuses on the contribution of voltage-dependent anion channel (VDAC) to cell life and death, and highlights its activity in relation to diseases such as cancer, Huntington disease, Alzheimer disease, diabetes, and heart disease.

Mammals, including humans, have three isoforms of VDAC, VDAC1, VDAC2, and VDAC3. These isoforms have very similar structure, conductance, and voltage-gating properties, but their existence points to VDAC functional complexity. While this research topic applies mainly to VDAC1, one of the topic papers presents a perspective on the evolution of VDAC function from molecular sieve to metabolic governator to actuator of ferroptosis. A second contribution reviews all VDAC isoforms with a focus on a unique role for VDAC3, in which cysteine oxidative modifications reflect the reactive oxygen species (ROS) load in the intermembrane space and mark oxidatively damaged mitochondria. Consequently, oxidative VDAC3 alterations may be an important element of different disease etiologies.

A third paper presents a comprehensive review of VDAC1 functions in mammalian cell cultures and animal models in health and disease. As the gatekeeper of the mitochondrion, VDAC1 in the outer membrane is crucially positioned as the principal interface between mitochondrial and cytosolic metabolism that controls cross-talk between mitochondria and the rest of the cell. VDAC1-linked functions also include regulation of apoptosis *via* release of pro-apoptotic proteins from the intermembrane space to the cytosol and interactions with apoptosis regulatory proteins, such as Bcl-2 family members and hexokinase. Finally, VDAC1 also contributes to structural and functional interactions between mitochondria and endoplasmic reticulum particularly to Ca^2+^ signaling. Indeed, VDAC1 is a docking site and hub protein interacting with over 100 proteins.

A fourth contribution addresses how free dimeric tubulin closes VDAC1 and VDAC2 to contribute to suppression of mitochondrial metabolism in the Warburg metabolic phenotype of aerobic glycolysis characteristic of cancer cells. Opening of these VDACs by small molecules increases mitochondrial metabolism and ROS generation as an anti-Warburg metabolic switch that leads ultimately to cell death.

The fifth research topic paper describes the possible contribution of VDAC1 to Huntington disease. The presented data indicate that the first measurable mitochondrial change after mutant huntingtin (mHtt) expression in an inducible cell model of the disease is an elimination of basal respiration fluctuations observed in the presence of the wild-type protein (Htt). Such elimination co-occurs with a decrease in the viability of cells expressing mHtt and a higher frequency of VDAC, principally VDAC1, transition into the closed state. Interestingly, the effect may result from VDAC–tubulin interaction-based mechanisms proposed to exist in cancer cells.

To conclude, VDAC, as a convergence point for a variety of both cell survival and death signals, can be regarded as a rational target for developing VDAC-based cytoprotective and cytotoxic strategies for treating diseases triggered or mediated by mitochondria dysfunction and damage. Indeed, as reviewed in the third topic paper, various strategies involving RNA interference to downregulate VDAC1 expression as well as activation of apoptosis *via* VDAC1-based peptides and inhibition of VDAC1 conductance by small molecules were developed and validated in several cancer mouse models., Thus, this research topic reveals VDAC isoforms as proteins important for proper cell function and highlights possible VDAC1-based new avenues in metabolism- and apoptosis-oriented human disease treatments.

## Author Contributions

All authors listed have made a substantial, direct, and intellectual contribution to the work and approved it for publication.

## Conflict of Interest Statement

The authors declare that the research was conducted in the absence of any commercial or financial relationships that could be construed as a potential conflict of interest.

